# Histone post-translational modification and heterochromatin alterations in neurodegeneration: revealing novel disease pathways and potential therapeutics

**DOI:** 10.3389/fnmol.2024.1456052

**Published:** 2024-09-13

**Authors:** Raven M. A. Fisher, Mariana P. Torrente

**Affiliations:** ^1^PhD. Program in Biochemistry, City University of New York - The Graduate Center, New York, NY, United States; ^2^Department of Chemistry and Biochemistry, Brooklyn College, Brooklyn, NY, United States; ^3^PhD. Programs in Chemistry, Biochemistry, and Biology, City University of New York - The Graduate Center, New York, NY, United States

**Keywords:** histone post translational modification, epigenetics, neurodegeneration, heterochromatin, chromatin

## Abstract

Alzheimer’s disease (AD), Parkinson’s disease (PD), Frontotemporal Dementia (FTD), and Amyotrophic lateral sclerosis (ALS) are complex and fatal neurodegenerative diseases. While current treatments for these diseases do alleviate some symptoms, there is an imperative need for novel treatments able to stop their progression. For all of these ailments, most cases occur sporadically and have no known genetic cause. Only a small percentage of patients bear known mutations which occur in a multitude of genes. Hence, it is clear that genetic factors alone do not explain disease occurrence. Chromatin, a DNA-histone complex whose basic unit is the nucleosome, is divided into euchromatin, an open form accessible to the transcriptional machinery, and heterochromatin, which is closed and transcriptionally inactive. Protruding out of the nucleosome, histone tails undergo post-translational modifications (PTMs) including methylation, acetylation, and phosphorylation which occur at specific residues and are connected to different chromatin structural states and regulate access to transcriptional machinery. Epigenetic mechanisms, including histone PTMs and changes in chromatin structure, could help explain neurodegenerative disease processes and illuminate novel treatment targets. Recent research has revealed that changes in histone PTMs and heterochromatin loss or gain are connected to neurodegeneration. Here, we review evidence for epigenetic changes occurring in AD, PD, and FTD/ALS. We focus specifically on alterations in the histone PTMs landscape, changes in the expression of histone modifying enzymes and chromatin remodelers as well as the consequences of these changes in heterochromatin structure. We also highlight the potential for epigenetic therapies in neurodegenerative disease treatment. Given their reversibility and pharmacological accessibility, epigenetic mechanisms provide a promising avenue for novel treatments. Altogether, these findings underscore the need for thorough characterization of epigenetic mechanisms and chromatin structure in neurodegeneration.

## Introduction

As human life spans have continued to increase over the last few decades, neurodegenerative diseases have dramatically risen in prevalence (Checkoway et al., 2011). Alzheimer’s disease (AD), Parkinson’s disease (PD), Frontotemporal dementia (FTD), and Amyotrophic lateral sclerosis (ALS) are major neurodegenerative disorders (NDs) affecting different neuron types and leading to a plethora of symptoms. Interestingly, in all these disorders, the majority of cases are considered sporadic and are presumed to arise from environmental factors either acting alone or in conjunction with various dysregulated genes (Spencer et al., 2022; Dilliott et al., 2021). A small proportion of cases -often termed familial- are linked to genetic mutations occurring in a large number of genes. Notably, in many instances, these genetic mutations lead to the misfolding and aggregation of specific proteins such as amyloid precursor protein (*APP*) in AD, α -synuclein (*SNCA*) in PD and Chromosome 9 open reading frame 72 (*C9orf72*) in FTD/ALS (Schellenberg and Montine, 2012; Stefanis, 2012; Balendra and Isaacs, 2018). As genetics alone does not explain disease etiology, epigenetics could reveal pathological mechanisms and novel treatment avenues.

Epigenetics involves heritable alterations to phenotype that do not result from a change in DNA sequence (Shahid et al., 2023). Eukaryotic DNA folds and condenses itself around a histone protein octamer consisting of two H2A-H2B dimers and a H3–H4 tetramer (Jenuwein and Allis, 2001). This DNA and histone complex constitutes the nucleosome, the basic repeating unit of chromatin (Shahid et al., 2023; Jenuwein and Allis, 2001; Chereji et al., 2018). In addition to DNA methylation and non-coding RNA action, epigenetic mechanisms include histone post-translational modifications as well as chromatin remodeling, invoking changes in nucleosomal positioning and chromatin structure (Hwang et al., 2017; Bannister and Kouzarides, 2011).

Protruding out of the nucleosome, the tails of histone proteins can undergo various post-translational modifications (PTMs) including acetylation, methylation, and phosphorylation as well as ubiquitylation and SUMOylation (Bannister and Kouzarides, 2011). Other modifications such as mono-ADP-ribosylation, formylation, citrullination, and crotonylation are also possible. Although these other modifications are categorized, functional roles and mechanisms are not fully understood (Zhao and Garcia, 2015). Histone modifications impact DNA-templated processes not only by modulating the physical interaction between histones and DNA but also by serving as binding platforms for other proteins (Shahid et al., 2023; Jenuwein and Allis, 2001). In this way, PTMs comprise a ‘code’ through which they largely regulate gene transcription. Each modification is site-specific and connected to distinct functions. For example, methylation can be linked to transcriptional repression or activation depending on which site is modified (Jenuwein and Allis, 2001; Peterson and Laniel, 2004). Furthermore, ubiquitylation and SUMOylation have also been linked to gene silencing, transcriptional repression as well as DNA repair at certain sites (Bannister and Kouzarides, 2011; Zhao and Garcia, 2015). Accordingly, the enzymes responsible for adding, removing, and recognizing these PTMs are referred to as the writers, erasers, and readers of this code, respectively (Rothbart and Strahl, 2014). For instance, acetylation marks are written by histone acetyltransferases (HATs) such as Gcn5/PCAF, erased by histone deacetylases (HDAC) such as HDAC 1, and read by proteins that contain bromodomains such as BRD4 (Marmorstein and Trievel, 2009; Saha and Pahan, 2006; Marmorstein and Zhou, 2014). Similarly, methylation marks are written by histone methyltransferases (HMTs) like SET7, erased by histone demethylases such as Lysine Specific Demethylase 1 (LSD1), and read by proteins comprising chromodomains such as CHD1 (Teperino et al., 2010; Yun et al., 2011). Phosphorylation marks are written by kinases such as Aurora B Kinase, erased by phosphatases such as Repo-man/PP1, and read by phospho-serine adaptor molecules like 14–3-3 proteins (Peterson and Laniel, 2004; Gil and Vagnarelli, 2019; Winter et al., 2008). One well-characterized ubiquitylation mark, H2AK119ub, is linked to gene repression (Bannister and Kouzarides, 2011). This modification is written by ubiquitin ligase, RING1B, and erased by deubiquitinase, BAP1 (Zhao and Garcia, 2015; Barbour et al., 2020).

Chromatin typically assumes two states which are defined by their accessibility to transcriptional machinery and are directly linked to particular histone PTMs: euchromatin and heterochromatin. On one hand, euchromatin is open and transcriptionally active; it is linked to acetylation at Lysine 9 and 14 (H3K9ac and H3K14ac) as well as phosphorylation at Serine 10 on H3 (H3S10ph) specifically during interphase (Torrente et al., 2011; Komar and Juszczynski, 2020). Euchromatin is typically found within active *cis*-regulatory elements such as promoters and enhancers (Morrison and Thakur, 2021).

Conversely, heterochromatin is closed, compact, and transcriptionally silent (Shahid et al., 2023). It is further divided into either constitutive heterochromatin or facultative heterochromatin. Facultative heterochromatin (fHC) assembles around highly regulated genes during development and is cell type-specific (Penagos-Puig and Furlan-Magaril, 2020). fHC is characterized by the presence of polycomb group proteins and tri-methylation of Histone H3 at Lysine 27 (H3K27me3) as well as monoubiquitylation of H2A at Lysine 119 (H2AK119ub) (Morrison and Thakur, 2021). H3K27me3 is installed by polycomb repressive complex 2 (PRC2), a set-domain containing methyltransferase (Millán-Zambrano et al., 2022; Laugesen et al., 2016). PRC2 subunits EED and SUZ12 allow the protein complex to also bind to H3K27me3 and propagate fHC. This mark is removed by various demethylases such as KDM6A, KDM6C, and KDM6B (Hyun et al., 2017). Additionally, H2AK119 ubiquitination is catalyzed by polycomb repressive complex 1 (PRC1) which has the ability to phase separate and can cause chromatin compaction without H2Aub (Barbour et al., 2020; Eeftens et al., 2021). Monoubiquitylation of H2A is removed by the deubiquitinase, BAP1 (Barbour et al., 2020). One noteworthy reader of H2AK119ub, JARID2, promotes PRC2 methylation of H3K27me3 linking these two fHC PTMs in a positive feedback mechanism (Barbour et al., 2020; Eeftens et al., 2021).

Constitutive heterochromatin (cHC) comprises a static condensed state that is conserved in different cell types and propagated throughout life once it is established (Morrison and Thakur, 2021). Tri-methylation on H3 at Lysine 9 (H3K9me3) is a key mark characterizing cHC. H3K9me3 is installed by methyltransferases SUV39H1 and SUV39H2. JHDM3/KDM4 are responsible for demethylating this mark (Hyun et al., 2017). Heterochromatin protein 1 (HP1), which binds H3K9me3, is necessary for constitutive heterochromatin condensation. HP1 can compact chromatin into phase-separated liquid condensates (Larson et al., 2017). Acting opposite to H3K9me3, phosphorylation of H3 at Serine 10 (H3S10ph) occludes HP1 binding to H3K9me3 and ejects HP1 from chromatin in a “binary switch” mechanism (Heinrichs, 2005). Indeed, H3S10ph is a key factor in cell cycle regulation and chromatin dynamics due to its ability to kick out HP1 during mitosis (Komar and Juszczynski, 2020).

In conjunction with chromatin, nuclear lamina is necessary for nuclear shape and DNA integrity. Nuclear lamina is found within the nuclear envelope and is comprised of intermediate filament proteins known as Lamin (Carollo and Barra, 2023). Due to their interaction with chromatin, Lamin proteins are important genomic organization factors. Nuclear Lamins, enriched in Lamin proteins B1 and B2, interact with specific chromosomal regions known as Lamin-associated domains (LADs) which are typically consistent with heterochromatin regions (Carollo and Barra, 2023; Maji et al., 2020; van Steensel and Belmont, 2017). Lamin B1 decreases are indicative of heterochromatin decreases (Stephens et al., 2018). In this way, Lamins are thoroughly connected to heterochromatin and indirectly to histone PTMs. Interestingly, Lamin tail domains binding to H2A and H2B are evolutionarily conserved (Goldberg et al., 1999). Alterations in Lamin expression and protein levels, histone PTMs, and chromatin lead to abnormal nuclear morphology and nuclear blebs which are connected to a multitude of diseases (Stephens et al., 2018).

Recent evidence points to the dysregulation of histone PTMs and alterations in heterochromatin playing a notable role in ND pathways (Wang et al., 2021; Song et al., 2023; Lee et al., 2018; Bennett et al., 2019; Chen et al., 2018; Kabir et al., 2023; Gräff et al., 2012; Zimmer-Bensch, 2020; Cobos et al., 2019). Methylation, acetylation, phosphorylation, and ubiquitylation of histone tails have thoroughly been implicated in various ND’s. The role of other modifications such as citrullination, formylation, and crotonylation is yet to be fully understood in this context. Histone PTMs are an attractive drug target given their dynamic nature and accessibility to pharmaceutical intervention. Here, we review changes on the histone PTM and heterochromatin landscape in AD, PD, and FTD/ALS and underscore the potential for novel neurodegeneration therapeutics targeting these epigenetic mechanisms. These findings highlight the need for comprehensive characterization of epigenetic mechanisms and chromatin structure alterations in ND research. We note that while aging and environmental exposures are also linked to epigenetics alterations and ND occurrence (Mir et al., 2023), we will not discuss these factors here. Instead, for an in-depth exploration of these topics, we refer the reader to several thorough reviews (Hou et al., 2019; Lee J.-H. et al., 2020; Cannon and Greenamyre, 2011; Chin-Chan et al., 2015).

## Histone PTM and heterochromatin changes in AD

Alzheimer’s disease (AD) is a neurodegenerative disease characterized by neuritic plaques and neurofibrillary tangles in the medial temporal lobe and neocortical parts of the brain (Breijyeh and Karaman, 2020). AD has been long associated with misfolding and aggregation of amyloid-beta peptides and Tau proteins (Penney et al., 2020; Gerrish et al., 2012; Bloom, 2014). Most cases of AD are sporadic, for which aging is the greatest risk factor, and occur later in life than the rarer familial forms (Bali et al., 2012; Lozupone et al., 2023). Hence, genetic mutations do not explain most AD cases. Furthermore, the pathological mechanisms by which amyloid-beta and Tau aggregation lead to neurodegeneration remain unknown. Mounting evidence implicates epigenetic mechanisms in AD. Below, we survey recent findings connecting sporadic AD as well as Amyloid-beta and Tau AD to epigenetic mechanisms and heterochromatin structure.

### Sporadic AD

Histone PTMs are connected to neural plasticity and a variety of other important neuronal factors (Farrelly and Maze, 2019). Highlighting a potential link to AD, histone acetylation is linked to synaptic plasticity and memory formation (Guan et al., 2009; Guan et al., 2002). Furthermore, recent evidence links epigenetic alterations to AD cellular dysfunction suggesting a pathological role for epigenetic mechanisms. For instance, cortical neurons derived from sporadic AD-induced pluripotent stem cells (iPSC) displayed reduced *BMI1* mRNA and protein levels (Flamier et al., 2018). *BMI1* is a major component of PRC1, a H2AK119ub ubiquitin ligase complex connected to fHC (Gray et al., 2016). Interestingly, *BMI1* levels were unaffected in familial AD models suggesting this change is independent of amyloid and tau toxicity (Flamier et al., 2018). BMI1 forms a complex with RING1, an E3 ligase subunit of PRC1, and is necessary for enzymatic function. The exact role of BMI1 in the PRC1 complex is not fully understood (Gray et al., 2016). Additionally, temporal cortices from sporadic AD brains displayed increased H3K9me3 levels leading to altered expression of *BDNF* (Brain-derived neurotrophic factor), *HIST2H2BE* (Histone H2B type 2E), and *SYT12* (Synaptotagmin 12). BDNF and SYT12 mRNA levels were significantly reduced while HIST2H2BE mRNA levels were increased (Lee M. Y. et al., 2020). Histone variant H2BE is typically expressed in olfactory neurons (Santoro and Dulac, 2012). Notably, olfactory impairment and dysfunction, which could be connected to overexpression of H2BE, have been considered early indicators of neurodegeneration (Bhatia-Dey and Heinbockel, 2021). Lastly, ATAC-seq and RNA-seq studies on A*POE* carriers, a main genetic risk factor for age-linked sporadic AD, revealed overall increased chromatin accessibility within peripheral immune cells when compared to healthy controls (Ramakrishnan et al., 2024). Together, the reduction of BMI1 expression in cortical neurons and increased chromatin accessibility in peripheral immune cells of APOE carriers implicate heterochromatin changes in sporadic AD. Decreases in BMI1 levels coupled with increases in H3K9me3 levels suggest fHC decondensation and cHC hypercondensation, respectively (Flamier et al., 2018; Lee M. Y. et al., 2020). Overall, these results suggest that heterochromatin alterations are associated with sporadic AD pathology. However, additional research is needed to definitively establish this association.

### Amyloid beta

Amyloid-beta (A β) is a component of the amyloid precursor protein (APP) and plays a key role in the pathogenesis of AD (Chen et al., 2017; Hampel et al., 2021). Mutations in APP lead to A β protein aggregation, misfolding, and plaque formation (Hampel et al., 2021). A β mutations have been connected to changes in the epigenetic landscape. In APP mice models, the euchromatic marks H4K12ac and H4K5ac are repressed by APP leading to downregulation of immediate early genes, *Egr1*, *c-Fos*, and *Bdnf*. These genes are involved in memory formation, neuronal plasticity, and neuronal growth and survival, respectively (Hendrickx et al., 2014). Of note, inhibition of BRD4, an acetylation reader, increased A β levels in H4 cells expressing APP, indicating a role for histone acetylation in A β pathology (Zhang et al., 2022). BRD4 has a bromodomain which enables it to bind to H4K5ac through a conserved mechanism that is not yet fully understood (Marmorstein and Zhou, 2014). Moreover, in mice hippocampal neurons, treatment with amyloid-beta oligomers (A β ) increased HDAC2 levels (Gräff et al., 2012). HDACs remove acetyl groups and the negative charge from histones which promotes chromatin condensation (Teijido and Cacabelos, 2018). Interestingly, HDAC2 is responsible for deacetylating H4K5 and H4K12 further implicating Histone H4 acetylation in the pathological mechanism of A β (Hendrickx et al., 2014). In another study involving mice models overexpressing mutant APP, H3K14ac and H3K9me2 levels were increased in the neocortices of the brain linking increased transcriptional activity with A β toxicity. Furthermore, both histone hypermethylation and hyperacetylation were found in adult murine neurons overexpressing A β (Lithner et al., 2013). Additionally, H3K9ac levels were decreased in *S. cerevisiae* overexpressing amyloid- β 1–40 (Hugais et al., 2021). H3K9ac has been implicated in memory formation and its decrease is common in aging brains (Currais et al., 2019). Lastly, increased H3S10ph levels were found in the cytoplasm of AD patient neurons compared to patient controls, providing a link between cell cycle re-entry and AD (Ogawa et al., 2003). Overall, A β overexpression and APP mutation are associated with variations in histone acetylation and methylation levels. Increased H3K14ac, H3S10ph, and H3K9me2 appear to counteract each other’s transcriptional effect but ultimately indicate genomic instability and suggest heterochromatin loss (Zheng et al., 2019; Booth and Brunet, 2016). Downregulation of Egr1, c-Fos, and BDNF suggests decreased transcriptional activity at specific genomic loci, while epigenetic changes suggest global heterochromatin loss. Interestingly, hypoacetylation of H4 coupled with increased HDAC2 expression suggests a connection between A β pathology and DNA replication. Aside from playing a role in transcription, acetylation of H4 has been linked to chromatin decompaction during DNA replication (Vettese-Dadey et al., 1996; Ruan et al., 2015). Further studies looking at distinct cell types and specific genetic loci will illustrate the difference between local and global epigenetic alterations. While these observations provide strong evidence for a link between A β pathology and epigenetics, further research is needed to uncover functional and mechanistic details.

### Tau

Mutations in the MAPT gene, encoding for the Tau protein, are thoroughly implicated in various neurodegenerative diseases including AD (Gerrish et al., 2012; Leveille et al., 2021). These diseases have been categorized as tauopathies as Tau aggregation is a major feature of their disease mechanism (Leveille et al., 2021). Tau -a microtubule-associated protein- becomes hyperphosphorylated and causes neurofibrillary tangles in AD (Maina et al., 2018). Tau acts in a concerted manner with A β where aggregation of A β leads to Tau hyperphosphorylation and mislocalization of Tau leads to increases in A β (Leveille et al., 2021). Several lines of evidence tie Tau to chromatin processes. Remarkably, Tau has been found to localize with cHC and be necessary for cHC stability (Sjöberg et al., 2006). Additionally, in SH-SY5Y cells, the knockdown of Tau led to decreased H3K9me2 and H3K9me3 levels further supporting a link between Tau expression and cHC stability (Maina et al., 2018). In murine neurons, the knockout of Tau protein causes disrupted H3K9me3 levels within chromocenters, interphase-occurring heterochromatin dense masses (Mansuroglu et al., 2016; Gerbi, 2018). In the same study, AD brains displayed an altered distribution of H3K9me3 in the presence of pathological phosphorylated Tau (pTau) showing that pTau causes the same epigenetic response as Tau protein loss (Mansuroglu et al., 2016; El Hajjar et al., 2019). Additionally, in postmortem AD patient brains and Tau mice models, the euchromatic mark H3K4me3 was significantly increased leading to the upregulation of genes commonly implicated in neurodegeneration such as *Sgk1* (serum and glucocorticoid-regulated kinase 1) which is involved in memory formation and neuronal plasticity (Cao et al., 2020; Lang et al., 2010). Levels of the methyltransferases KMT2C/2D and SETD1A/1B were found also to be increased in AD excitatory neurons (Cao et al., 2020). Histone methyltransferases (HMTs) catalyze the transfer of methyl groups from (*S*-adenosylmethionine) SAM to lysine or arginine residues. Among HMTs, lysine methyltransferases (KMTs) are site-specific and contain SET domains (Bannister and Kouzarides, 2011). Increased H3K4me3 KMTs and increased PTM levels suggest that heterochromatin loss is linked to Tau AD.

**Figure 1:**
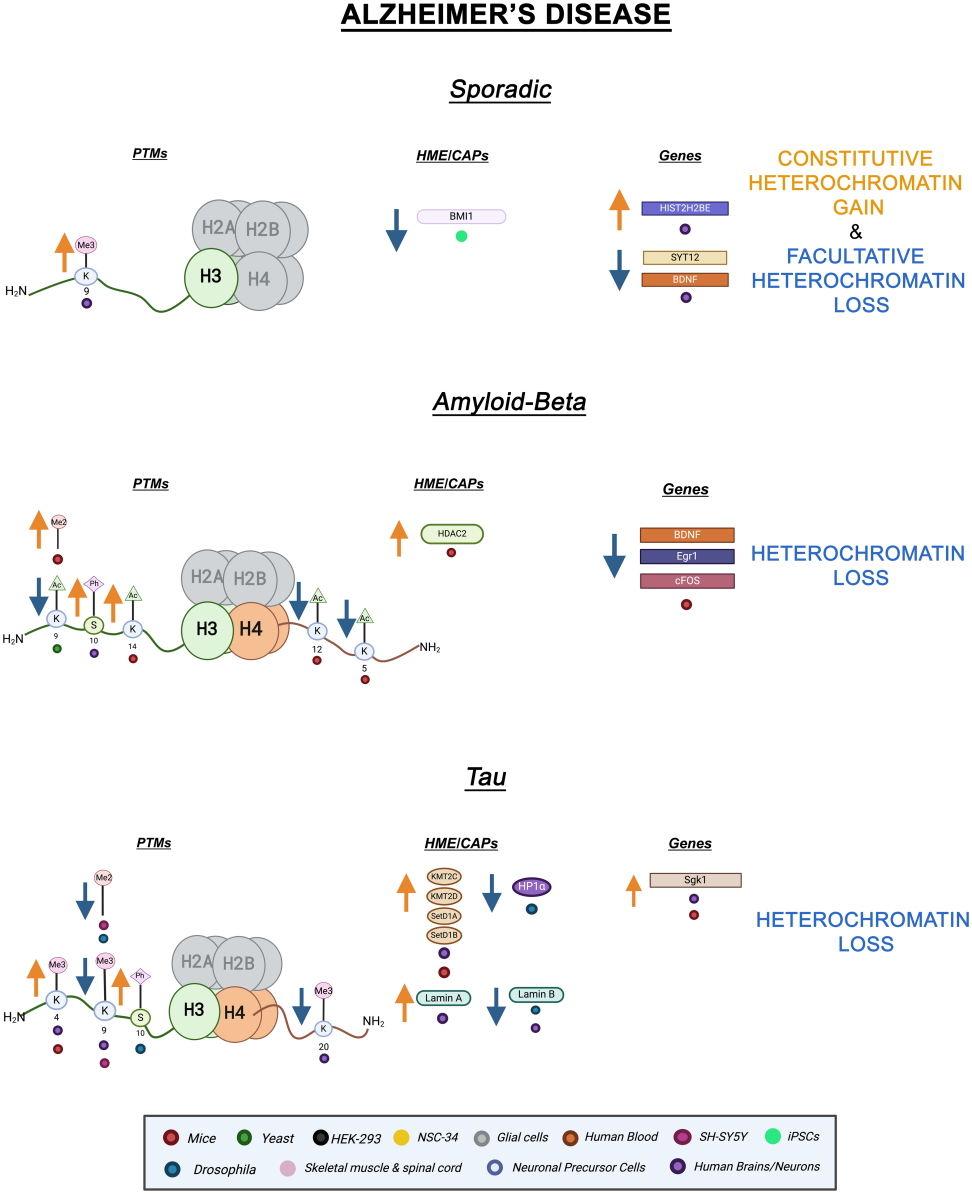
Epigenetic alterations linked to sporadic and familial AD. Columns indicate histone post-translational modifications (PTMs), genes, histone-modifying enzymes (HMEs), and chromatin-associated proteins (CAPs) altered in AD. Yellow arrows denote increases in the levels of proteins, genes, or histone post-translational modifications, while blue arrows denote protein, gene, or post-translational modification level decreases. Colored dots indicate the model system as depicted in the legend. Global Heterochromatin gain is indicated in yellow font, while global heterochromatin loss is indicated in blue font. Figure created with Biorender.com.

Tau AD has also been connected to Lamin dysfunction. In *Drosophila* expressing disease-associated Tau mutants, Lamin protein levels were decreased leading to altered nuclear morphology (Frost et al., 2016). Moreover, *Drosophila* expressing a loss of function *Lam* allele recapitulated features of pathological Tau including decreased H3K9me2 and HP1 α levels (Frost et al., 2016; Frost et al., 2014). In AD patient brains, Lamin B protein levels were decreased (Frost et al., 2016). In contrast, AD patient hippocampal neurons from all disease stages displayed high expression of Lamin A while Lamin B2 was dysregulated by nucleoplasm accumulation (Gil et al., 2020). In agreement with the notion that high levels of Lamin A are pathological, lower levels of Lamin A and mRNA levels of its respective gene, LMNA, are hypothesized to be protective against AD pathology (Méndez-López et al., 2019). Additionally, heterochromatin-dense chromocenters are lost in *Lam* mutant *Drosophila* neurons and AD patient brains (El Hajjar et al., 2019). The same study revealed increased H3S10ph levels in *Drosophila* neurons by immunostaining (Frost et al., 2016). Overall, Lamin dysfunction -induced either by mutation or pathogenic Tau- leads to epigenetic alterations suggesting heterochromatin loss. Decreased repressive PTMs and increased active PTMs occurring in AD indicate reduced heterochromatin stability. Tau pathology is specifically linked to cHC changes. Tau toxicity connects to decreases in Lamin and repressive histone PTM levels which play an important role in AD pathogenesis. Additional epigenetic investigations are imperative in this context. A graphic summary of the epigenetic alterations in AD discussed here is presented in Figure 1.

#### Therapeutic applications in AD

Although AD is the most common neurodegenerative disease, there is no cure; recent treatments, although now able to slow disease progression, still produce adverse effects (Knopman and Hershey, 2023). Older therapies including acetylcholinesterase inhibitors such as Donepezil and Rivastigmine aid in improving impaired cognition (Breijyeh and Karaman, 2020). New drugs such as Lecanemab, an anti-amyloid antibody, have shown success with amyloid removal in clinical trials (Knopman and Hershey, 2023). Donanemab, another anti-amyloid antibody, has slowed disease progression in clinical trials but does not reverse disease progression. Interestingly, Donanemab is more effective in patients with low Tau levels indicating a need for Tau-targeting therapies (Reardon, 2024). Despite these advances, there is room for improvement in AD treatment. Epigenetic therapies could provide great benefits to patients in the treatment and even prevention of AD.

Potential epigenetic therapies for AD are already under investigation. Targeting histone methyltransferases has been recently explored as a treatment for many neurodegenerative diseases and cancer (Marzochi et al., 2023). Treating Tau mutant mice and 5xFAD mice with WDR5-0103, a SET1/MLL family HMT inhibitor, restored H3K4me3 levels as well as synaptic function and memory (Cao et al., 2020). Additionally, treatment of FAD mice with the EHMT1/2 inhibitor, BIX01294, ameliorated memory loss and restored H3K9me2 levels (Zheng et al., 2019).

As dysregulation of acetylation marks is a key part of AD pathology, histone deacetylase inhibitors (HDACi) are some of the most promising novel epigenetic treatments. Some HDACis are already used therapeutically in other diseases such as cancer (Eckschlager et al., 2017). HDACIs lead to increased levels of histone acetylation which decreases the positive charge on histones and loosens their association with the negatively charged DNA backbone. This leads to increased chromatin accessibility and transcriptional activity (Carrier, 2013; Gallinari et al., 2007). Multiple studies in mice have shown the benefits of treatment with HDAC inhibitors. Although AD is associated with epigenetic changes mostly consistent with global heterochromatin loss, HDACIs can restore downregulated genes due to local decreases in transcription. For example, treatment of murine APP+/+ cortical neurons with the HDAC inhibitor Trichostatin (TSA) overcomes the inhibitory effects of APP and induces the transcription of immediate early gene *Egr1*, early growth response gene necessary for synaptic plasticity and long-term potentiation (Hendrickx et al., 2013). Moreover, treatment of APP/PS1 mice with BG45, another class I HDACi, led to decreased phosphorylated Tau expression and neuronal loss (Han et al., 2022). Another study in triple transgenic AD mice and primary cortical neurons treated with the HDACi Sulforaphane showed enhanced expression of BDNF and increased global H3 and H4 acetylation levels (Kim et al., 2017). Lastly, in presenilin 1 and 2 double knockout mice, treatment with the HDACi sodium butyrate reduced Tau hyperphosphorylation. This treatment also resulted in increased H3 acetylation and alleviated loss of contextual memory (Cao et al., 2018). In ApoE3/E4 patient astrocytes, a sporadic AD model, treatment with class I HDACis MS275 and CI994 increased mRNA expression of apoE and increased astrocytic protein secretion (Dresselhaus et al., 2018). While these findings are encouraging, HDAC inhibitors tend to lack selectivity and hence cause undesirable side effects. Of note, most epigenetic-targeting drugs -termed epidrugs- produce off-target effects due to a lack of specificity (Feehley et al., 2023). The development of highly selective and potent HDAC inhibitors with minimal off-target effects is required for the successful translation of these to the clinic (Yang et al., 2017). Combinations of different epidrugs can aid in overcoming adverse effects (Feehley et al., 2023; Majchrzak-Celińska et al., 2021). Nevertheless, HDAC inhibition remains a promising strategy for AD therapy.

## Histone PTM and heterochromatin changes in PD

Parkinson’s disease (PD) is a progressive neurodegenerative disorder affecting dopaminergic neurons in the substantia nigra (Iarkov et al., 2020). PD usually manifests with tremors and motor problems such as muscular rigidity and gait instability (Song et al., 2023). Much like AD, PD is mostly sporadic with influences from complex environmental factors such as stress and aging (Chai and Lim, 2013). Familial PD is linked to mutations in many genes including SNCA and PRKN which encode for α-synuclein ( α -SYN) and Parkin, respectively (Stefanis, 2012; Castelo Rueda et al., 2021). Akin to AD, the mechanisms linking protein aggregation to cell death remain incompletely characterized. Epigenetic factors have been thoroughly implicated in all forms of PD. Chromatin processes could potentially reveal yet undiscovered PD mechanisms.

### Sporadic PD

Aside from environmental factors such as heavy metal exposure and drug use (Ball et al., 2019), sporadic PD has been associated with certain mutations in genes such as SNCA and LRRK2 (leucine-rich repeat kinase 2) (Chai and Lim, 2013; Tolosa et al., 2020). SETD1A, a H3K4 methyltransferase implicated in neurodevelopmental and neuropsychiatric disorders, has been identified as a sporadic PD risk gene but its pathological involvement is not yet understood (Spataro et al., 2015; Witoelar et al., 2017; Chong et al., 2022; Booms et al., 2024). In sporadic PD iPSC-derived dopaminergic neurons, H3K4me3 – a euchromatin mark- is enriched at the SNCA promoter (Guhathakurta et al., 2021). Moreover, in sporadic PD neuronal precursor cells (NPCs) and dopaminergic neurons, mitochondrial dysfunction leads to the reduction of H3K9me3 and H3K27me3 levels due to metabolic dysregulation (Schmidt et al., 2023). In peripheral whole blood cells of sporadic PD patients, transcriptomics revealed differential expression of CBX5, the gene encoding HP1 α . CBX5 mRNA levels were decreased in PD patients when compared to healthy controls. Expression of HP1’s interacting partner, DNMT3A, and additional chromatin interacting proteins such as methyltransferase, Enhancer of zeste homolog 1 (Ezh1) were also decreased in PD patient blood cells (Calligaris et al., 2015). Furthermore, in a sporadic PD-like mice model, *En1*+/−, there was a reduction in DAPI-dense regions of heterochromatin, and a decrease in heterochromatin marks H3K27me3 and H3K9me3 (Rekaik et al., 2015).

As in AD, PD is linked to Lamin, but this link is not yet fully understood. In sporadic PD, substantia nigra pars compacta astrocytes displayed reduced nuclear Lamin B1 protein levels (Chinta et al., 2018). Decreased Lamin levels suggest a connection between heterochromatin instability and PD. Overall, these alterations implicate heterochromatin loss, both cHC and fHC, in sporadic PD pathology.

### α -SYN

α -SYN is a member of the synuclein protein family which predominately functions as neuronal proteins, localizing at the presynaptic terminals (Stefanis, 2012). Mutations in SNCA lead to misfolding and aggregation of α -SYN (Stefanis, 2012; Song et al., 2023). These aggregates form Lewy bodies, cytoplasmic inclusions found in the substantia nigra that are a hallmark of PD (Song et al., 2023; Gomperts, 2016). α -SYN overexpression has been connected to changes in histone methylation and heterochromatin composition. Remarkably, both transgenic *Drosophila* and SH-SY5Y α -SYN overexpression models display enhanced H3K9me2 levels and HP1 α expression (Sugeno et al., 2016). In the same study, α -SYN expression increased EHMT2 and SUV39H2 (H3K9 methyltransferases) levels while EZH1 (H3K27 methyltransferase homolog of EZH2) levels were slightly decreased (Sugeno et al., 2016; Margueron et al., 2008). Additionally, differentiated SH-SY5Y cells overexpressing α -SYN displayed increased protein levels of lysine demethylases KDM1B, KDM4B, and KDM4C which target H3K4 and H3K9 methylation, respectively (Hyun et al., 2017; Sugeno et al., 2016). Histone demethylases (HDMs) have different catalytic mechanisms and specificity. Interestingly, KDM1 (LSD1) mechanism utilizes amine oxidation to demethylate H3K4 mono- and di-methylated residues. It is also inactive against trimethylated residues. On the other hand, KDM4B (JMJD2B) undergoes hydrogen bonding within its binding pocket and exhibits specificity for H3K9 di and tri-methylation (Marmorstein and Trievel, 2009). The site and modification specificity of HDMs makes them an attractive epigenetic target.

α -SYN expression has also been linked to disturbances in histone acetylation. In murine dopaminergic neurons, cytosolic α -SYN expression reduces the activity of histone acetyltransferase p300 (Jin et al., 2011). Interestingly, p300 is responsible for acetylating a variety of proteins, including Smad1, a major intracellular effector of Bone morphogenic proteins (BMP), which play a key role in neurogenesis (Pearson et al., 1999). HATs utilize different catalytic mechanisms depending on cellular context. Of note, distinctly from other HATs, p300 uses a tyrosine residue for acid catalysis (Marmorstein and Trievel, 2009). The variation in mechanisms of histone acetylation may allow for enhanced specificity for epigenetic drugs. Furthermore, in *S. cerevisiae* overexpressing α -SYN, H3K36me2, and H2BT129ph levels are significantly decreased. In yeast, these PTMs are responsible for transcriptional elongation and DNA damage, respectively (Chen et al., 2018). Altogether, these findings show a link between α -SYN expression histone modification and heterochromatin dysregulation in various PD models. Increased repressive PTMs and methyltransferase levels as well as decreased activating PTMs suggest heterochromatin gain, specifically cHC, in α -SYN pathology. Nonetheless, this has not been directly shown yet.

### Parkin

**Figure 2:**
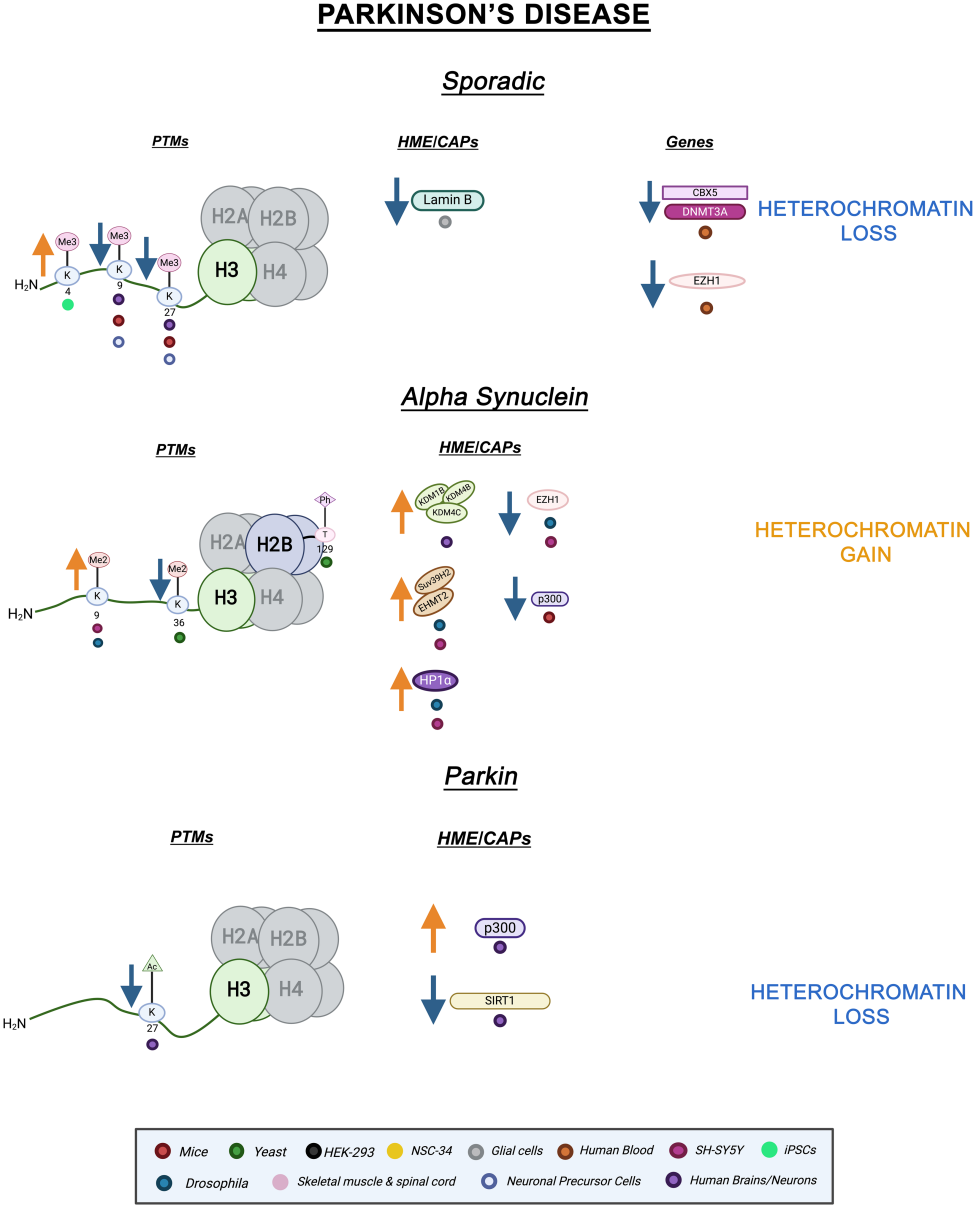
Epigenetic alterations are linked to sporadic and familial PD. Columns indicate histone post-translational modifications (PTMs), genes, histone-modifying enzymes (HMEs) and chromatin-associated proteins (CAPs) altered in PD. Columns are omitted when no changes are revealed. Yellow arrows denote increases in the levels of proteins, genes, or histone post-translational modifications, while blue arrows denote protein, gene, or post-translational modification level decreases. Colored dots indicate the model system as depicted in the legend. Global heterochromatin gain is indicated in yellow font, while global heterochromatin loss is indicated in blue font. Figure created with Biorender.com.

Parkin, a ubiquitin ligase, is implicated in various cellular processes such as inflammation and mitochondrial biogenesis (Castelo Rueda et al., 2021). Mutations in the gene, PRKN, are the most common cause of early-onset PD (Castelo Rueda et al., 2023). Parkin also has neuroprotective properties and regulates the accumulation of α -SYN (Jęśko et al., 2019). The role of epigenetic factors such as miRNA and DNA methylation have been explored within the context of PRKN mutations, but histone PTMs and chromatin structure changes remain mostly uncharacterized (Tsalenchuk et al., 2023). Emerging evidence points to a role for histone PTMs in Parkin PD. For instance, genome-wide hyperacetylation of H3K27 coupled to a decrease in class III HDAC, SIRT1, activity and increased histone acetyltransferase, p300, activity were revealed within the PRKN gene in PD brains (Toker et al., 2021; Milazzo et al., 2020). Histone hypoacetylation and decreased p300 activity have been found in SNCA PD models as well as other neurodegenerative diseases (Beaver et al., 2020; Valor et al., 2013). Both hypo- and hyperacetylation can alter homeostasis and proteostasis leading to disease pathology (Kabir et al., 2023). PRKN-linked hyperacetylation on H3K27 suggests an increase in transcriptional activation and a loss of fHC, hinting at an important mechanistic role for H3K27ac in PD pathology. Figure 2 illustrates the epigenetic alterations linked to familial and sporadic PD discussed here.

#### Therapeutic applications in PD

Akin to AD, PD lacks an effective cure. To promote dopamine synthesis, treatment of PD currently involves the dopamine precursor, L-DOPA. Unfortunately, L-DOPA leads to side effects such as motor complications, emotional and psychiatric disturbances (Fox and Lang, 2008; Salat and Tolosa, 2013). For those reasons, novel treatments and preventative measures for PD are quite needed.

As expression of α -SYN is modulated by histone methylation, this modification is an attractive therapeutic target (Guhathakurta et al., 2021). As mentioned previously, KMTs and KDMs are already in the clinic for cancer therapy (Marzochi et al., 2023; Morera et al., 2016). Treating SH-SY5Y cells overexpressing α -SYN with the histone-lysine n-methyltransferase 2 (EHMT2) inhibitor, UNC0638, restored expression levels of L1CAM and Snap25, genes repressed by increased H3K9me2 (Sugeno et al., 2016). Treatment of PD mice with GSK-J4, a histone demethylase inhibitor able to cross the blood–brain barrier, rescued H3K27me3 alterations conferring neuroprotective effects. SH-SY5Y cells also display increased H3K27me3 after GSK-J4 treatment (Mu et al., 2020). Furthermore, in sporadic PD brains and iPSC models, CRISPR-Cas9 recruited Jarid1A, a histone demethylase, was able to reduce α -SYN, and restore H3K4me3 levels at the SNCA locus (Guhathakurta et al., 2021).

HDACi treatment ameliorates α -SYN toxicity. Both transgenic *Drosophila* and SH-SY5Y cells treated with sodium butyrate displayed reduced α -SYN toxicity (Kontopoulos et al., 2006). Histone acetyltransferase (HAT) modulators -activators of p300 specifically- could alleviate the reduction of p300 activity triggered by α -SYN expression (Jin et al., 2011); however, these compounds are not yet ready for clinical translation due to poor solubility and permeability (Teijido and Cacabelos, 2018; Jin et al., 2011). Epigenetic treatments remain unexplored for PRKN-associated PD. Nevertheless, the alterations in histone post-translational modifications reviewed above suggest promising therapeutic targets in this context.

## Histone PTM and heterochromatin changes in FTD/ALS

Frontotemporal dementia (FTD) is characterized by degeneration of neurons in the frontal and temporal lobes of the brain. One of the most common dementias, FTD manifests with personality and behavioral changes as well as language difficulties (Khan and De Jesus, 2023). FTD exists on a disease continuum with amyotrophic lateral sclerosis (ALS), a neurodegenerative disease affecting motor neurons throughout the motor cortex, brainstem, and spinal cord (Mead et al., 2023). ALS symptoms typically include progressive muscular weakness (Abramzon et al., 2020). FTD/ALS has clinical and pathological overlap with patients developing symptoms attributed to either disease or both diseases (Cividini et al., 2022).

Much like other neurodegenerative diseases, most cases of FTD/ALS are sporadic. Familial FTD/ALS is linked to mutations in many genes (Abramzon et al., 2020). *SOD1*, *TARDBP*, *FUS*, and *C9orf72* are some of the key genes implicated in FTD/ALS. Mutations in each of these genes lead to aggregation and misfolding of their respective proteins (Gendron et al., 2013; Schwartz et al., 2014; Starr and Sattler, 2018). A growing body of data links epigenetic mechanisms, including histone PTM and heterochromatin alterations, to FTD/ALS pathology.

### Sporadic FTD/ALS

Epigenetic mechanisms are associated with both sporadic and familial FTD/ALS (Paez-Colasante et al., 2015). For instance, peripheral blood mononuclear cells (PBMCs) from sporadic ALS patients displayed altered ATAC-seq profiles revealing increased chromatin accessibility compared to healthy controls. The differentially accessible genes were enriched in those involved in neuronal health and function (Kühlwein et al., 2023). In sporadic ALS postmortem brains and spinal cords, HDAC2 mRNA was significantly increased while HDAC11 mRNA was decreased (Janssen et al., 2010). HDAC2 is a class I HDAC that can deacetylate lysine residues on histone tails and non-histone targets. On the other hand, HDAC11 is a class IV HDAC associated with antigen-specific T-cell response and is not as understood as the other HDACs (Gallinari et al., 2007; Milazzo et al., 2020; Janssen et al., 2010). Increased HDAC2 and decreased HDAC11 suggest reduced transcriptional accessibility and immune response, respectively (Janssen et al., 2010). Interestingly, post-mortem sporadic ALS spinal motor neurons showed depleted chromatin remodeling complex Brg1/Brm Associated Factor (BAF). The BAF complex interacts with acetylated histones through its tandem PHD finger domain (Bannister and Kouzarides, 2011). BAF loss was also observed in familial and sporadic cases with C9orf72 mutations hinting at its importance in disease processes (Tibshirani et al., 2017). DNA methylation changes were observed in sporadic FTD, but histone PTMs and chromatin structure have not been thoroughly examined in this context (Galimberti et al., 2013). Overall, while there is evidence for heterochromatin loss in sporadic ALS, additional research is needed to definitively ascertain this feature (Fisher et al., 2023).

### SOD1

Mutations in the Cu/Zn superoxide dismutase-1 (*SOD1*) gene is one of the most common mutations in both familial and sporadic ALS (Parobkova and Matej, 2021). These mutations lead to protein aggregation and mitochondrial mislocalization, but the exact pathological mechanism is not yet understood (Parobkova and Matej, 2021; Berdyński et al., 2022). SH-SY5Y cells expressing SOD1 mutants G93A and H80R as well as SOD1 G39A transgenic mice showed decreased H3S10ph-K14ac, a combinatory euchromatic mark, and H3K4me2, another modification connected to gene transcription. Importantly, the reduction in the levels of H3S10ph-K14ac was directly linked to increases in SOD1 expression (Masala et al., 2018). In another study using the same SOD1 murine model, DNMT3A – a DNA methylase – levels were reduced in both skeletal muscle and spinal cord mitochondria (Wong et al., 2013). Of note, HP1𝛼 has long been known to interact and associate with DNMTs presumably linking SOD1 proteinopathy with heterochromatin alterations (Fuks et al., 2003). Furthermore, in SH-SY5Y cells overexpressing SOD1 G93A, mutant SOD1 was found to strongly associate with chromatin. While wild-type SOD1 also interacts with chromatin, mutant SOD1 has higher affinity suggesting interactions with DNA and nucleosomes are increased by pathogenic mutations (Barbosa et al., 2010). Lastly, NSC-34 cells overexpressing SOD1 G93A showed increased immunoreactivity and protein levels of lysine-specific histone demethylase-1 (LSD1) and decreased immunoreactivity and protein levels of H3K4me2 (Choi et al., 2022). Altogether, overexpression of pathogenic SOD1 mutants revealed decreases in transcriptionally active histone PTMs, H3S10ph-K14ac, and H3K4me2 suggesting a role for heterochromatin gain and loss of transcriptional activity within SOD1 pathology. These results lay the foundation for further epigenetic studies in SOD1 models that will definitively link heterochromatin changes to SOD1 pathology.

### TDP-43

Mutations in *TARDBP*, which encodes for TDP-43 -a gene expression regulator- lead to protein mislocalization, dysfunction, and nuclear depletion (Gendron et al., 2013; Yan et al., 2014; Liu et al., 2019). The overwhelming majority of FTD/ALS cases, including both sporadic and familial cases, display TDP-43 inclusions (Fisher et al., 2023; Ducharme et al., 2024). In a TDP-43 mutant murine model, TDP-43 cytoplasmic overexpression led to differential expression and aberrant polyadenylation of histones. Canonical histone genes were upregulated while variant histone genes were downregulated (Amlie-Wolf et al., 2015). Downregulated histone variants suggest the prevention of critical histone-swapping events (Henikoff and Smith, 2015). Interestingly, in the frontal cortex of FTD patients cytoplasmic TDP-43 aggregates led to reduced levels of CHD2, an ATP-dependent chromatin remodeling factor. Additionally, in the same study, HEK293 cells overexpressing TDP-43 displayed reduced nucleosome clearance and decreased expression of various heat shock genes such as HSPA1A and HSPA6, suggesting a role for TDP-43 in regulating chromatin dynamics (Berson et al., 2017). Furthermore, in FTD murine models, TDP-43 pathogenesis has been linked to reduced HDAC1 (class I deacetylase) activity (Han et al., 2022; Wu et al., 2020). Our own work has revealed histone PTM perturbations in yeast FTD/ALS models. In yeast overexpressing TDP-43, H3K36me3 levels decrease, while H4K12ac and H4K16ac levels increase (Chen et al., 2018). As previously mentioned, methylation of H3K36 is involved in transcriptional elongation as well as chromatin structural maintenance of coding regions while H4K16ac is involved in transcriptional activation. Together, H3K36me3 decreases and H4K16ac increases are indicative of enhanced transcriptional activity and decreased chromatin stability (Chen et al., 2018). Altogether, TDP-43 proteinopathy leads to alterations in histone PTMs and chromatin modifiers indicative of genomic instability and heterochromatin breakdown.

### FUS

Mutations in *FUS*, an RNA-binding protein, are also notably implicated in FTD/ALS. Cytoplasmic and nuclear FUS aggregates occur in the motor and cortical neurons of FTD/ALS patients (Schwartz et al., 2014; Mackenzie et al., 2011). Mislocalization of nuclear FUS into cytoplasmic inclusions has been linked to epigenetic dysregulation in FTD/ALS. In mice motor neurons, when FUS mislocalizes to the cytoplasm, protein arginine methyltransferase 1 (PRMT1) is depleted from the nucleus. PRMT1 is responsible for di-methylating arginine 3 on histone H4 (H4R3me2) which is a mark typically associated with transcriptional activity (Tibshirani et al., 2015). Arginine methyltransferases utilize similar catalytic mechanisms to lysine methyltransferases. Interestingly, these enzymes exhibit some specificity in how SAM and the histone come in contact with the catalytic site. This suggests selectivity that can be helpful for epigenetic targeting (Bannister and Kouzarides, 2011). In the same study, H4R3me2 was revealed to be decreased in motor neurons displaying cytoplasmic FUS. Combinatory marks H3K9ac-K14ac were also decreased (Tibshirani et al., 2015). Loss of these transcriptionally active PTMs, especially H4R3me2, has been thoroughly implicated in chromatin hypercondensation and increased heterochromatin formation (Huang et al., 2005). In yeast overexpressing FUS, we find decreases in H3K14ac, H3K56ac, H4R3me2, and H3S10ph levels which are all linked to transcriptional activation (Chen et al., 2018). Additionally, H3K56ac plays a role in DNA replication and genomic stability therefore its decrease would link FUS mislocalization with genomic instability and errors in replication (Yuan et al., 2009). Overall, pathogenic FUS leads to decreases in activation-linked PTMs and histone-interacting enzymes. These findings suggest a connection between FUS pathology, transcriptional silencing, and potentially heterochromatin gain.

### C9orf72

C9orf72, the most common genetic cause of FTD/ALS, pathologically includes a hexanucleotide repeat expansion (G_4_C_2_) which leads to neurodegeneration through several mechanisms (Zhu et al., 2020). One such mechanism invokes the production of five toxic dipeptide repeat proteins (DPRs) via repeat-associated non-AUG translation: proline-alanine (PA), glycine-proline (GP), glycine–alanine (GA), glycine-arginine (GR), and proline-arginine (PR) (Taylor et al., 2016). DPRs aggregate and ultimately cause neuronal death (Freibaum and Taylor, 2017).

Expanding evidence links DPR pathology with epigenetic alterations. Histone trimethylation causes reduced *C9orf72* mRNA levels in both the frontal cortex and cerebella of c9 FTD/ALS patients. Of note, mutant *C9orf72* gene binding to H3K9me3 and H3K27me3 was detected in the blood of C9 FTD/ALS patients (Belzil et al., 2013). This suggests epigenetic alterations could be useful as diagnostic biomarkers. Frontal cortices and cerebellum of repeat expansion carriers also revealed increased H3K79me3 (Belzil et al., 2013).

Remarkably, heterochromatin alterations have been directly linked to poly PR toxicity. Upon poly PR expression in mice, this DPR localizes to heterochromatin and causes an increase in both the heterochromatic mark H3K27me3 and the euchromatic mark H3K4me3. Remarkably, poly PR also decreases HP1 α expression in mice and alters HP1 phase separation properties *in vitro* (Zhang et al., 2019). Additionally, C9BAC mice astrocytes show depletion of the heterochromatin mark H3K9me3 (Jury et al., 2020). Very recently, epigenomic studies of c9ALS patients have revealed increased H3K27ac, an activating histone mark. Furthermore, c9ALS patients displayed overall increased chromatin accessibility in non-neuronal cells such as glia and astrocytes at distal regions of differentially expressed ALS genes, such as ERGIC1 and MYOE1, suggesting heterochromatin decondensation (Li et al., 2023). In c9FTD/ALS patient brains, EZH2 expression was decreased when compared to healthy controls. Notably, EZH2 was revealed to form insoluble aggregates in c9 brains suggesting a mechanism for decreased protein levels (Wang et al., 2019). In C9orf72 *Drosophila*, C9 HRE’s lead to redistribution of proteins due to errors in nucleocytoplasmic transport (Ortega et al., 2020). Histones were found to be enriched in the nucleus as expected but interestingly, PRMT1 was cytosolically enriched evidencing problems with nuclear transport (Ortega et al., 2020; Herrmann and Fackelmayer, 2009). Conversely to AD and PD, altered nuclear morphology due to changes in Lamin expression and invaginations are not pathological in c9FTD/ALS. In C9 FTD/ALS iPSCs, nuclear Lamin B1 invaginations were revealed to occur with age, independently of C9 HREs, and occur similarly in healthy and disease samples (Coyne and Rothstein, 2021).

**Figure 3:**
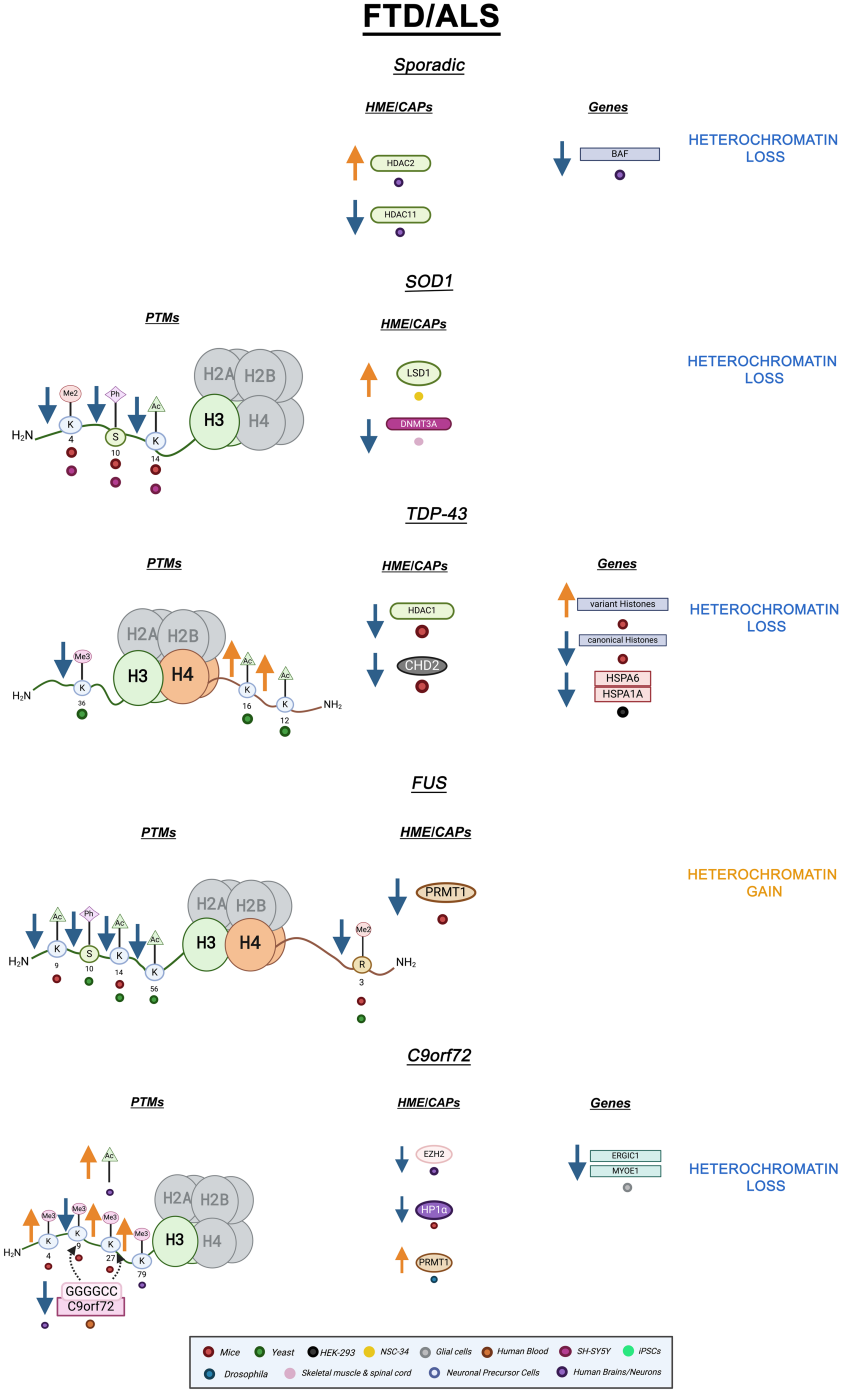
Epigenetic alterations are linked to sporadic and familial FTD/ALS. Columns indicate histone post-translational modifications (PTMs), genes, histone-modifying enzymes (HMEs), and chromatin-associated proteins (CAPs) altered in PD. Columns are omitted when no changes are revealed. Yellow arrows denote increases in the levels of proteins, genes, or histone post-translational modifications, while blue arrows denote protein, gene, or post-translational modification level decreases. Black arrows denote binding interactions. Colored dots indicate the model system as depicted in the legend. Global heterochromatin gain is indicated in yellow font, while global heterochromatin loss is indicated in blue font. Figure created with Biorender.com.

All in all, changes in active and repressive PTMs coupled with disruption of HP1 and EZH2 suggest heterochromatin decondensation is a key factor in c9FTD/ALS. These results suggest cHC loss but fHC involvement should not be ruled out. EZH2 and Suz12, subunits of PRC2, are necessary for HP1 α stability and might affect cHC formation and propagation (Boros et al., 2014). Altogether, both hypercondensation and decondensation of heterochromatin are implicated in FTD/ALS depending on gene mutation. A graphic summary of the epigenetic alterations in FTD/ALS covered in this review is presented in Figure 3. Additionally, Table 1 summarizes all the ND-relevant epigenetic factors including histone-modifying enzymes and chromatin remodeling complexes presented here.

**Table 1:** Histone interacting proteins involved in AD, PD, and FTD/ALS.

Disease	Proteinopathy	Epigenetic factors	Mechanism	Histone code role	Dysregulation	Model	Ref.
AD	Sporadic	BMI1	Component of ubiquitin ligase complex, PRC1	Writer	Decreased mRNA and protein levels	iPSCs	Flamier et al. (2018)
	Amyloid Beta	HDAC 2	Class I deacetylase	Eraser	Increased protein levels	Patient Brains	Gräff et al. (2012)
	Tau	KMT2C/2D	Lysine methyltransferase	Writer	Increased protein levels	Mice neurons	Cao et al. (2020)
		SETD1A/1B	Lysine methyltransferase	Writer	Increased protein levels	Patient neurons	
PD	Sporadic	CBX5	Encodes for HP1, interacts with H3K9me3	Reader	Decreased mRNA	Patient blood cells	Calligaris et al. (2015)
		EZH1	Lysine methyltransferase, homolog of EZH2	Writer			
	Alpha synuclein	HP1𝛼	Constitutive Heterochromatin Protein, Interacts with H3K9me3	Reader	Increased protein levels	Drosophila SH-SY5Y	Sugeno et al. (2016)
		EHMT2	H3K9me1/2 methyltransferase	Writer	Increased protein levels		
		SUV39H2	H3K9me3 methyltransferase	Writer	Increased protein levels		
		EZH1	Lysine methyltransferase, homolog of EZH2	Writer	Decreased protein levels		
		KDM1B/4B/4C	H3K4me1/2 and H3K9me2/3 demethylase	Eraser	Increased protein levels	SH-SY5Y	
		P300	Histone acetyltransferase	Writer	Reduced activity	Mice neurons	Jin et al. (2011)
	Parkin	SIRT1	Class III deacetylase	Eraser	Reduced activity	Patient Brains	Toker et al. (2021)
		P300	Histone acetyltransferase	Writer	Increased activity		
FTD/ALS	Sporadic	HDAC2	Class I deacetylase	Eraser	Increased mRNA	Patient brains and spinal cords	Janssen et al. (2010)
		HDAC11	Class IV deacetylase	Eraser			
		BAF	Chromatin Remodeler	Reader	Decreased protein levels	Patient neurons	Tibshirani et al. (2017)
		LSD1	H3K4me1/2 demethylase	Eraser	Increased immunoreactivity and protein levels	NSC-34	Choi et al. (2022)
	TDP-43	CHD2	ATP-dependent Chromatin Remodeler	Reader	Decreased protein levels	Patient brains	Berson et al. (2017)
		HDAC1	Class I deacetylase	Eraser	Reduced activity	Mice	Wu et al. (2020)
	FUS	PRMT1	H4R3me2 arginine methyltransferase	Writer	Decreased nuclear levels	Mice neurons	Tibshirani et al. (2015)
	C9orf72	HP1𝛼	Constitutive Heterochromatin Protein, Interacts with H3K9me3	Reader	Decreased expression and altered phase separation	Mice	Zhang et al. (2019)
		EZH2	H3K27me3 methyltransferase, enzymatic subunit of PRC2	Writer	Decreased protein levels and formation of insoluble aggregates	Patient brains	Wang et al. (2019)
		PRMT1	H4R3me2 arginine methyltransferase	Writer	Depleted from nucleus and cytosolically enriched	Drosophila	Ortega et al. (2020)

#### Therapeutic applications in FTD/ALS

Akin to other NDs, FTD/ALS is incurable but there are a few treatments available. Edaravone is an intravenous drug approved for ALS known to work against oxidative stress. It is found to be most effective during the early stages of the disease (Cho and Shukla, 2020). Riluzole, an FDA-approved ALS treatment, has provided patients with a slight increase in survival rates. Tofersen, an antisense oligonucleotide gene therapy, significantly reduces SOD1 mutant proteins in motor neurons and slows disease progression making it a very promising new therapy (Saini and Chawla, 2024). For FTD, antidepressants such as trazodone and NMDA receptor antagonists are typically used as symptomatic treatments (Mollah et al., 2024). Recent promising therapies for FTD include Latozinemab, targeting *GRN*-FTD, another FTD-associated gene, reduced progranulin levels (Ward et al., 2024; Crescioli et al., 2024). Although improved therapies have been developed in the last few years, treatment options able to stop FTD/ALS progression are still desperately needed (Mead et al., 2023).

Epigenetic treatments have begun to be utilized in FTD/ALS. Sodium phenylbutyrate is a histone deacetylase inhibitor which improves dysregulation of histone acetylation levels after treatment (Cudkowicz et al., 2009). Approved by the FDA in 2023 as a treatment for ALS, Relyvrio contains sodium phenylbutyrate and taurursodiol and reduces neuronal death in experimental models and clinical trials with only gastrointestinal problems as side effects (Beninger, 2023; Paganoni et al., 2020). However, it was pulled from the market in 2024 due to a lack of efficacy. Curiously, epigenetic channels are not thought to be involved in its mechanism of action. By restoring histone acetylation levels, TSA bypasses the toxic effect of FUS aggregation in yeast (Bennett et al., 2021). Additionally, TSA treatment improved motor neuron survival in SOD1 mice (Yoo and Ko, 2011). In SH-SY5Y cells expressing mutant TDP-43, treatment with the HDACis 4-phenylbutyrate, TSA, and sodium butyrate all increased cell survival and decreased TDP-43 toxicity (Sanna et al., 2020). Transgenic FUS+/+ mice treated with the HDACi ACY-738 displayed extended life, slowed disease progression, and restored H3K9ac and H3K14ac levels (Rossaert et al., 2019).

In SOD1 mice models, treatment with spermidine led to changes in chromatin structure and remodeling as well as changes in gene expression by targeting lysine-specific histone demethylase 1 (LSD1), the demethylase responsible for removing methyl groups from H3K4me2, leading to prolonged survival (Choi et al., 2022). Lastly, treatment with an inhibitor of BRD4 -bromodomain containing histone acetylation reader- JQ1, was shown to reduce cytotoxicity of poly-PR by blocking nuclear aggregation and leading to histone cytoplasmic accumulation in Hela cells treated with (PR)_20_ (Chen et al., 2024). Targeting epigenetic factors provides promising drug targets to restore altered histone acetylation and methylation and restore the expression of several histone-interacting proteins. These results highlight the importance of the inclusion of epigenetic mechanisms in neurodegeneration research. Further interrogation of these mechanisms will allow for discovery of attractive targets for chemical interference within these complex disease pathways. Epigenetic drugs remain a promising avenue to alter pathological manifestations of NDs that will hopefully halt disease progression. Table 2 summarizes all epigenetic treatments included in this review.

**Table 2:** Potential epigenetic treatments for neurodegenerative diseases.

Disease	Proteinopathy	Treatment	Mechanism of action	Ref.
AD	ApoE (sporadic)	MS275	Class I HDACi’s increased expression of apoE and astrocytic protein secretion.	Dresselhaus et al. (2018)
		Cl994		

	Amyloid-Beta	BG45	Class I HDACi decreases phosphorylated tau and alleviates neuronal loss.	Han et al. (2022)
		Sulforaphane	HDACi restores histone acetylation.	Kim et al. (2017)
		BIX01294	HMT inhibitor ameliorates memory loss stores perturbed H3K9me2.	Zheng et al. (2019)
		Trichostatin A	Alleviates APP inhibitory effect induces transcription of Egr1.	Hendrickx et al. (2013)
		Sodium butyrate	HDACi decreases tau phosphorylation and restores histone acetylation.	Cao et al. (2018)
	Tau	WDR5-0103	HMT inhibitor restores perturbed H3K4me3, synaptic formations and memory.	Cao et al. (2020)
PD	Sporadic	Jard1A	HDM inhibitor reduces 𝛼-Syn levels Restores perturbed H3K4me3	Guhathakurta et al. (2021)
	Alpha synuclein	UNC0638	HMT inhibitor restores perturbed H3K9me1/me2 and expression of H3K9me3 target genes.	Sugeno et al. (2016)
		GSK-J4	KDM inhibitor restores perturbed H3K27me3.	Mu et al. (2020)
		Sodium butyrate	HDACi reduces 𝛼-Syn toxicity.	Kontopoulos et al. (2006)
FTD/ALS	SOD1	Trichostatin A	HDACi improves motor neuron survival and muscular atrophy.	Yoo and Ko (2011)
		Spermidine	Antioxidant targets lysine specific demethylase 1 and improves cellular survival.	Choi et al. (2022)
	TDP-43	4 phenylbutyrate	HDACi increases cellular survival and ameliorates TDP-43 toxicity.	Sanna et al. (2020)
		Trichostatin A		
		Sodium butyrate		
	FUS	Trichostatin A	HDACi restores perturbed histone acetylation and ameliorates FUS toxicity.	Bennett et al. (2021)
		ACY-738	HDACi increases life span, survival rate and restores motor neuron phenotypes.	Rossaert et al. (2019)
	C9orf72	JQ1	BRD4 inhibitor reduces cytotoxicity of Poly-PR	Chen et al. (2024)

## Discussion and concluding remarks

Changes to the epigenetic landscape including histone PTM perturbations, alterations in the expression of histone-modifying enzymes (HMEs) and chromatin remodelers, as well as heterochromatin loss and gain are all implicated in neurodegeneration and provide a multitude of epigenetic targets. Altogether, the findings reviewed here illuminate the complexity and importance of the epigenetic landscape in NDs. The changes in histone PTMs and HMEs linked to heterochromatin, whether it be a heterochromatic loss (decondensation) or gain (hypercondensation), occur in the context of different genetic mutations in AD, PD, and FTD/ALS. Loss of heterochromatin may allow for cell cycle reentry leading to neurodegeneration (Frost et al., 2014; Wu et al., 2024). Post-mitotic neurons in the brain re-enter the cell cycle and reveal increased expression of a variety of ND risk genes. Increased cell cycle re-entry is typically observed in AD and PD pathology and is not a consequence of aging, connecting this occurrence with ND pathways (Wu et al., 2024).

It is important to note that while certain NDs appear to be associated with global heterochromatin loss, global chromatin changes do not necessarily mirror local changes at specific genomic loci. Therefore, certain genes can be downregulated even when heterochromatin loss indicates global increased transcriptional activity. For example, in A β linked AD, decreased expression of BDNF, Egr1, and c-Fos occurs even though there is a global heterochromatin loss. Differential histone PTM changes may be occurring at specific gene loci. Epigenomic techniques such as ChIP-seq can give more insight by linking global changes to specific genes (Zheng et al., 2019). Additionally, it is also possible that differential gene expression linked to epigenomic changes occurs in different cell types. Single-cell epigenomic investigations can aid in uncovering localized changes and lead to the development of more precise therapies. Moreover, open chromatin caused by heterochromatin loss is connected to increased DNA damage which plays a role in cell cycle re-entry of neurons suggesting a link between heterochromatin loss and neurodegeneration (Kim and Tsai, 2009). Additionally, heterochromatin hypercondensation may contribute mechanistically through repression and downregulation of genes with important cellular roles (Lee M. Y. et al., 2020). As discussed here, both hypercondensation and decondensation are connected to pathological manifestation of these diseases.

Altogether, these findings underscore the crucial need for the inclusion of epigenetic and chromatin structure studies in neurodegeneration. Whether these epigenetic mechanisms are causative or downstream effects of disease pathology, they nevertheless provide a promising avenue for novel treatments to impact cellular outcomes and survival. Epigenetic targets are attractive because while they are heritable, they are also pharmaceutically accessible and reversible. Due to their dynamic nature, they also have the potential to be used in diagnostics (Kelly et al., 2010). In conjunction with epidrugs, epigenetic editing offers a promising novel therapeutic strategy. Due to its precision and specificity, epigenetic editing bypasses the off-target effects usually associated with epidrugs (Ueda et al., 2023). The development of CRISPR-Cas9 has increased the versatility of epigenetic editing allowing for more epigenetic targets (Ueda et al., 2023; Liu and Jaenisch, 2019; Nakamura et al., 2021). For example, CRISPR-Cas9 can potentially be used to study and target other epigenomic factors such as HP1 phase separation and epitranscriptomic alterations (Nakamura et al., 2021). In addition to CRISPR technologies, zinc finger proteins (ZFP) and TALENs approaches possess the ability to fuse with epigenetic modifiers and allow for site specificity (Ueda et al., 2023). Although epigenetic editing expands the reach of epigenetic therapies, there are still many important considerations. CRISPR/Cas9 systems can induce background mutations, R-loop formations, and even cleave DNA (Ueda et al., 2023). Further work is necessary to understand the implications of utilizing epigenetic editing and to develop better technologies with less off-target effects (Sasaki-Honda et al., 2023).

While the results reviewed here illustrate the importance of epigenetic mechanisms in disease, a multitude of questions still remain. Are heterochromatin and histone PTM changes linked to specific genetic mutations recapitulated within different cell types? Can heterochromatin and histone PTM alterations be utilized as both diagnostic biomarkers and therapeutic targets? Uncovering disease pathways will be pivotal in understanding how heterochromatin changes arise or whether these changes are linked to pathological manifestations. Additional investigations into epigenomic and transcriptomic profiles of different cell types such as glial cells and diverse neuronal subtypes will help paint a full picture. Furthermore, continued investigations in sporadic models will also aid in further uncovering the differences and similarities between sporadic and familial diseases. Although this research area is full of promise, a more precise understanding of the epigenetic mechanisms at play in neurodegeneration is necessary to develop improved, clinically applicable epigenetic therapies.
